# Human Gamma Oscillations during Slow Wave Sleep

**DOI:** 10.1371/journal.pone.0033477

**Published:** 2012-04-04

**Authors:** Mario Valderrama, Benoît Crépon, Vicente Botella-Soler, Jacques Martinerie, Dominique Hasboun, Catalina Alvarado-Rojas, Michel Baulac, Claude Adam, Vincent Navarro, Michel Le Van Quyen

**Affiliations:** 1 Centre de Recherche de l'Institut du Cerveau et de la Moelle épinière (CRICM), Institut National de la Santé et de la Recherche Médicale (INSERM) UMRS 975, Centre National de la Recherche Scientifique (CNRS) - UMR 7225, Université Pierre et Marie Curie (UPMC), Hôpital de la Pitié-Salpêtrière, Paris, France; 2 Departament de Física Teòrica and Instituto de Física Corpuscular (IFIC), Universitat de València - Consejo Superior de Investigaciones Científicas (CSIC), València, Spain; 3 Epilepsy Unit, Assistance publique - Hôpitaux de Paris (AP-HP), Groupe Hospitalier Pitié-Salpêtrière, Paris, France; 4 Departamento de Ingeniería Eléctrica y Electrónica, Universidad de Los Andes, Bogotá, Colombia; Indiana University, United States of America

## Abstract

Neocortical local field potentials have shown that gamma oscillations occur spontaneously during slow-wave sleep (SWS). At the macroscopic EEG level in the human brain, no evidences were reported so far. In this study, by using simultaneous scalp and intracranial EEG recordings in 20 epileptic subjects, we examined gamma oscillations in cerebral cortex during SWS. We report that gamma oscillations in low (30–50 Hz) and high (60–120 Hz) frequency bands recurrently emerged in all investigated regions and their amplitudes coincided with specific phases of the cortical slow wave. In most of the cases, multiple oscillatory bursts in different frequency bands from 30 to 120 Hz were correlated with positive peaks of scalp slow waves (“IN-phase” pattern), confirming previous animal findings. In addition, we report another gamma pattern that appears preferentially during the negative phase of the slow wave (“ANTI-phase” pattern). This new pattern presented dominant peaks in the high gamma range and was preferentially expressed in the temporal cortex. Finally, we found that the spatial coherence between cortical sites exhibiting gamma activities was local and fell off quickly when computed between distant sites. Overall, these results provide the first human evidences that gamma oscillations can be observed in macroscopic EEG recordings during sleep. They support the concept that these high-frequency activities might be associated with phasic increases of neural activity during slow oscillations. Such patterned activity in the sleeping brain could play a role in off-line processing of cortical networks.

## Introduction

When sleep reaches its deepest levels during slow wave sleep (SWS), the electroencephalogram (EEG) is dominated by globally coherent slow wave activity in the low frequency range (0.5–3.5 Hz) [1], [2]. Local field potentials (LFPs) studies in animals, moreover, have demonstrated that the power time course of these slow oscillations shows correlations to other frequency bands. According to Steriade and coworkers [3], the slow oscillations have the ability to trigger and group faster cortical oscillations like spindles (12–15 Hz) and high-frequency activities in the beta (from 15 to 25 Hz) or gamma range (from 30 to 120 Hz). Evidence for this coupling between slow waves and gamma oscillations comes from in vivo LFP recordings of the rodent and feline cortex [3]–[7] and it can be reproduced in vitro in acute brain slices [8], [9]. These experiments demonstrated that gamma oscillations occur preferentially over the active component of the slow wave (‘UP’ state) characterized by rhythmic cycles of synaptically mediated depolarization and disappear during the hyperpolarizing phase (‘DOWN’ state). A recent study with microelectrode LFPs in the human cortex has confirmed that gamma oscillations are strongly expressed during SWS and are reliably associated with a marked increase in local cellular discharges, suggesting that they were associated with cortical UP states [10], [11]. Nevertheless, although activities in the gamma-range have been observed at the scalp level during a variety of cognitive tasks [12], no evidences of a phasic expression of gamma activities during SWS in human macroscopic EEG recordings were reported so far. For example, Fell and colleagues [13] used the scalp EEG data during sleep and showed that sigma activity (12–16 Hz) is modulated by slow EEG oscillations. In another study, Molle and collaborators [14] found that grouping of spindles and beta oscillations are coincident with slow waves in human SWS. However, both studies didn't find a significant phasic modulation of the gamma activities (>30 Hz) by the slow waves. It was suggested that the resistive properties of the skull, muscle artifacts and the relative distance of scalp electrodes from deeper generators may make it difficult to observe an intracortical modulation of gamma activity during sleep. These drawbacks can nevertheless be overcome with the use of intracranial recordings which furthermore allow the analysis of short-range spatially coherent activities that are not promptly available with scalp recordings. In the present study, we examined the presence of gamma patterns during polysomnographically defined sleep states using intracranial EEG recordings, collected in parallel with macroscopic scalp EEG, of 20 subjects who required a clinical invasive evaluation for the treatment of their epilepsy. This relatively large sample size and broad spatial sampling in this study (with a total of 740 investigated intracranial electrode sites) provided an opportunity to evaluate the intracranial distribution of gamma activities over the whole cortex. Furthermore, the simultaneous recording of slow waves by scalp EEG allowed us to analyze different phasic modulations of gamma activities by global slow components.

## Results

### Gamma oscillations during SWS

**Figure 1:**
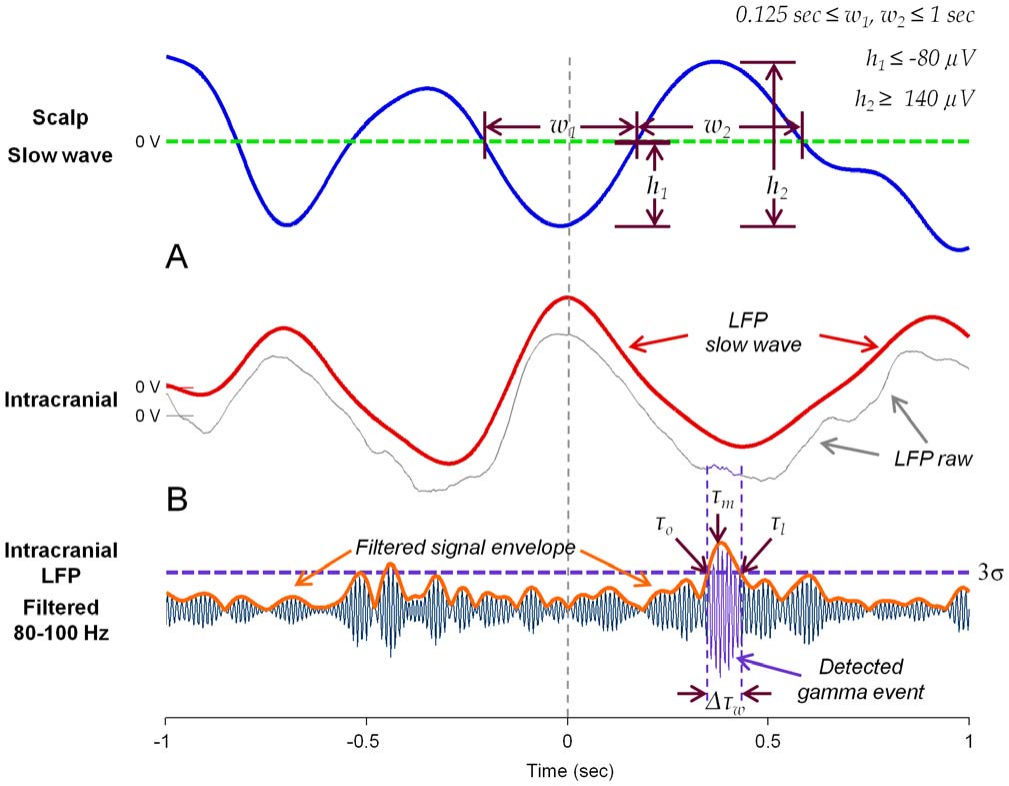
Automatic detection of slow waves and gamma events. (**A**) Examples of scalp (*blue line*) and intracranial (*red line*) detected slow waves, inside a 2-seconds window around the central peaks. Scalp slow wave were detected according to defined duration and amplitude criteria (*top right*). (**B**) Raw intracranial LFP (*gray line*) and the corresponding gamma event detected in the filtered LFP (*bottom*) when the amplitude of the envelope signal was over 3σ. Presented times and duration correspond to: *τ_o_*→first detection time, *τ_l_*→last detection time, *τ_m_*→maximal amplitude time, *Δτ_w_* = *τ_l_−τ_o_*→total event duration.

**Figure 2:**
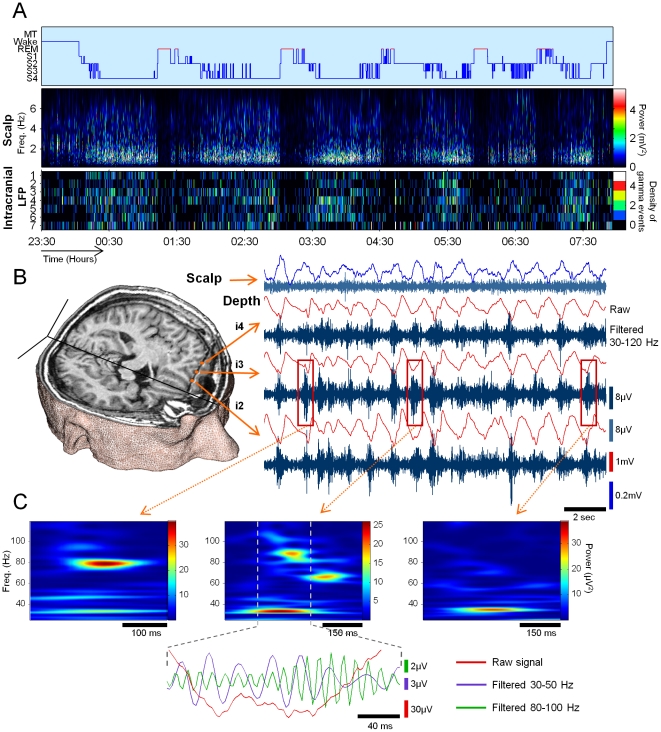
Gamma oscillations recorded with intracranial electrodes during SWS. (**A**) All-night hypnogram of a representative subject (*P15*) (*Top*), time-frequency evolution of the power for one scalp electrode (*Middle*), density of gamma events (30–120 Hz) detected on seven intracranial contacts located in the frontal and temporal cortex (*Bottom*). (**B**) 3D MRI reconstruction of the patient's brain presenting one depth electrode and its corresponding 6 intracranial contacts in the left orbito-frontal gyrus (*Left*). Simultaneous recordings during deep sleep (S4 stage) showing raw and 30–120 Hz filtered activities (presented below each raw one) for one scalp (FP1) and three depth contacts in the same cortical region (*Right*). (**C**) Time-frequency representations of the three bursts of gamma activity shown in (**B**) illustrating examples of pure oscillations with a narrow frequency in the high gamma range (*Left*), the low gamma range (*Right*), and complex oscillations composed of a few mixed low and high frequencies (*Middle*), as also shown in the filtered signals (*Bottom*).

A group of 20 epileptic subjects were included in this study. Intracranial LFPs were recorded from the surface of the cortex (subdurally) or from depth electrodes stereotactic implanted in deeper cortical structures. Details of electrode placement, location of interictal and ictal epileptic abnormalities are outlined in Table S1. All subjects had 2 selected overnight recordings. Episodes of gamma activity (30–120 Hz) were automatically identified across all stages of vigilance using previous methodology [15], and detected events were then visually confirmed in the raw traces (see *Materials and Methods* and Figure 1). In particular, for the majority of investigated intracranial contacts (79%, 227 of a total of 286; n = 8 subjects), the density of detected events (see below) was significantly higher during SWS than during wakefulness or REM (*p*<0.05, two-tailed permutation-test). A representative example of the gamma detection degree in relation to the sleep-wake cycle for a single subject is given in Figure 2*A*. As expected, the time-frequency representation of the power for a scalp electrode (Figure 2*A* *middle*) showed a clear increment of the power in the low frequency range (∼0.3–2 Hz) as the subject entered in the deepest stages of sleep (S2–S4). This increased rate of slow waves was in turn accompanied by an increment in the density of gamma events, measured as the number of detected events per time interval (20 seconds), presented here for seven intracranial contacts (Figure 2*A* *bottom*). In average, the mean density of gamma events during NREM sleep periods was 1.14±0.45 (mean ± SD) events per 20-seconds interval (n = 8 subjects), reflecting a large variability of gamma occurrences on each slow wave activity cycle.

In order to illustrate the relation between the gamma activity and the presence of slow waves, Figure 2*B* illustrates typical recorded patterns during sleep stage 4. A predominant slow activity can be seen in raw signals for both, surface (FP1) and depth electrodes (here in the left orbito-frontal gyrus). Note the reversed polarities between the scalp and the intracranial activities, confirming what has been previously shown in animal studies [3]. The average main peak in the power spectra was estimated here at 0.85±0.08 Hz (on average over n = 25 contacts) and the wave shapes at the scalp level fulfilled standard electrographic criteria of normal slow oscillations [16]. Concerning the signals filtered in the gamma-range, near-periodic bursts of increased amplitude could be observed in all intracranial contacts (but not at level of the scalp) at the depth-negative phase (respectively surface-positive phase) of the slow oscillations in the raw signals. A spectral analysis of the gamma envelopes indicated a main frequency peak of these bursts at 0.88±0.15 Hz (n = 25 contacts), confirming a relationship between the occurrences of gamma bursts and the slow oscillations during SWS. In either the raw signals (without any previous filtering or pre-processing) or those filtered between 30 and 120 Hz, fast sinusoidal waves appeared in all intracranial contacts as discrete events which were clearly distinguishable from background activity (Figure 2*C*, *bottom*). The wavelet transformed-energy scalogram, which represents the spectral energy with respect to time and frequency, precisely characterizes the frequency components of single detected gamma events. For an individual recording contact, successive cycles of the slow oscillation were constituted by a series of pure oscillatory bursts appearing in narrow frequency bands in the low (∼30–50 Hz) gamma range, high (∼60–120 Hz) gamma range or by broad-band events composed of a few mixed frequencies (Figure 2*C*). When broad-band events were observed, the different oscillatory components could be superimposed, slightly overlapping, or completely non-overlapping in time (Figure 2*C* *middle*). Note that gamma activity is plainly observed in the deepest intracranial contacts, suggesting that these activities cannot be explained by superficial scalp muscle artifacts, which are regardless very low during SWS.

Even though the intracranial electrodes in any individual subject explored a limited part of the brain, the relatively large sample size (20 subjects; see also Figure 6, yellow spheres) allowed us to analyze the anatomical distribution of gamma events. We found that gamma episodes were seen bilaterally during SWS in all investigated regions of the cerebral cortex (20/20 subjects). From all contacts located in the temporal lobe (451/740), 56% presented a significant higher density of gamma events during SWS than during other stages (comparing density during SWS vs. density in other stages together, p<0.05, two-tailed permutation-test). For the frontal lobe (142/740) and occipital lobe (76/740), higher detections occurred in 42% and 71% of all analyzed contacts, respectively. We found no significant correlations between the locations of gamma oscillations and the spatial extension of the seizure onset zone or propagation regions of epileptic spikes (58% of contacts presenting significant higher density of gamma oscillations during SWS were detected outside of these regions), suggesting that epilepsy is unlikely to be the main source of the observed oscillations.

### Phasic modulation of gamma oscillations by the slow waves

**Figure 3:**
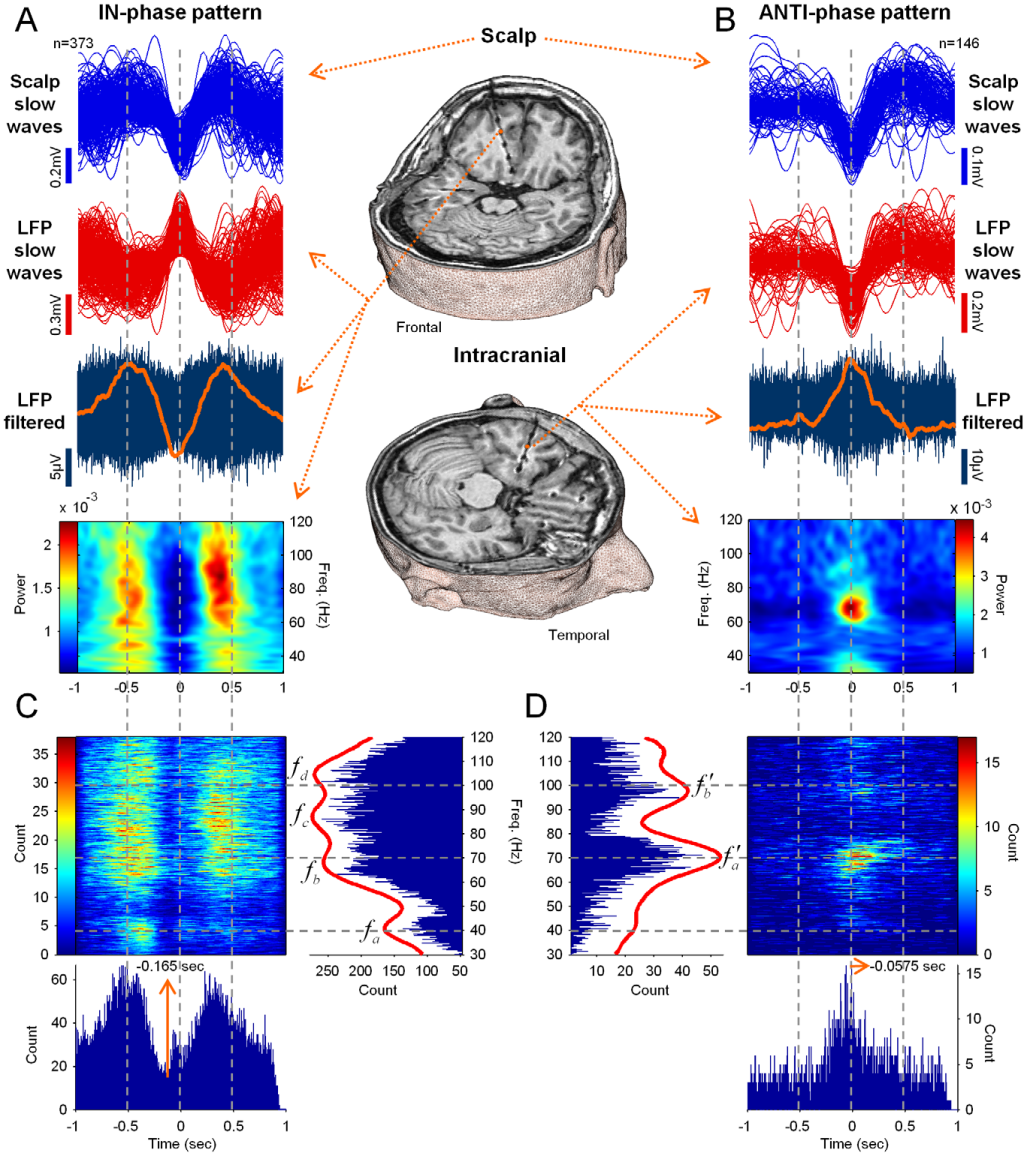
Two patterns of phasic modulation of gamma oscillations by the slow waves. (**A–B**) Examples of IN-phase and the ANTI-phase modulation patterns of gamma oscillations recorded in intracranial contacts in the cingulate gyrus (subject *P15*) (**A**) and the superior temporal sulcus (subject *P18*) (**B**), respectively. From *Top* to *Bottom*: Superimposed scalp slow waves aligned around their central peak; Simultaneously recorded intracranial LFPs, also showing slow components; Intracranial filtered (30–120 Hz) signals with the average RMS activity (orange line); Average of their time-frequency representations. (**C–D**) Total number of gamma events, associated with IN-phase and ANTI-phase patterns respectively, detected along frequency (30–120 Hz) and time (2-seconds window around the scalp negative peak) for all patients (n = 19) and all analyzed contacts (n = 233). For IN-phase and ANTI-phase patterns, the cumulative histograms of their frequency distributions were illustrated (*Top-Right* and *Top-Left*) with the corresponding regression curves (*red lines*). Several dominant peaks were identified at *f_a_*≈41, *f_b_*≈67, *f_c_*≈87, *f_d_*≈105 Hz and *f′_a_*≈70, *f′_b_*≈97 Hz. The cumulative histograms of their first detection times (relatives to the intracranial central peak of the slow waves) are also illustrated (*Bottom*).

To examine the possible modulation of gamma activity by the slow waves, we analyzed the temporal relationship between the phases of the slow wave and the occurrence of gamma activity. Over the entire recording session, the automatic detection algorithms independently identified slow waves and gamma oscillations for all investigated intracranial contacts (see *Materials and Methods*). Only slow waves where gamma events were detected inside a window of 2 seconds around the central peak of each intracranial slow wave were selected for analysis (see Figure 1 and Figure 3). Furthermore, we analyzed only contacts having a sufficient number of detected slow waves (>25; 233 of 740 contacts from 19 of 20 subjects). Analysis of individual gamma activities revealed three broad patterns of gamma modulation by the slow oscillation: In the first pattern, gamma events preferentially appeared during the positive phase of the scalp slow wave (“IN-phase” pattern). In the second, gamma events tend to appear during the negative phase of the scalp slow wave (“ANTI-phase” pattern). In the third pattern, there was no specific phase preference of the incidence of gamma events to the slow wave, meaning that gamma activity could be equally present during the positive as well as during the negative phase. Examples of phasic gamma modulations associated with IN-phase and ANTI-phase patterns are shown in Figure 3*A and 3B* for intracranial contacts in the cingulate gyrus and the superior temporal sulcus. For both patterns, the majority of gamma events were constituted by single oscillatory bursts lying in a narrow frequency band (63% and 62% of the cases for Figure 3*A* and Figure 3*B*, respectively; see also histograms in Figure S1). For the IN-phase pattern, however, a broad-band extension of activities in the gamma band can be identified as consequence of the overlapping, after averaging, of multiple oscillatory bursts appearing in different frequency bands across the whole gamma range from 30 to 120 Hz (Figure 3*A* *bottom*). In contrast, a narrow band extension of gamma activities around 70 Hz were associated with the ANTI-phase pattern (Figure 3*B* *bottom*). Additional examples of phasic gamma modulation associated with IN-phase and ANTI-phase patterns are illustrated in Figure 7*F*.

Over all subjects, gamma activities from all investigated electrodes were associated with one of the three patterns by using a measure of similarity between the distributions in time of the detected gamma events (see *Material and Methods*). From all selected gamma events, 52% of the intracranial contacts (122/233) from 15 of 19 subjects were modulated by the IN-phase pattern (Figure 3*C* *top-left*). In contrast, only 9% of the contacts (21/233) from 7 of 19 subjects (5 from the 15 above) were modulated by the ANTI-phase pattern (Figure 3*D* *top-right*). The total number of individual slow waves selected from all electrode contacts associated with the IN-phase and ANTI-phase patterns were 20363 and 2078 respectively. From these slow waves, gamma oscillations were, in most of the cases, constituted by pure narrow band frequencies for both, IN-phase and ANTI-phase patterns (56% and 60% of the cases respectively). Oscillatory bursts composed of two mixed frequencies were seen in 42% and 38% of each case. Concerning the distribution of their frequency, gamma events associated with the IN-phase pattern (Figure 3*C* *top-right*) had multiple dominant peaks scattered across the whole gamma range at around 41, 67, 87 and 105 Hz. For the ANTI-phase pattern, in contrast, only two dominant peaks could be seen in the high gamma range around 70 and 97 Hz (Figure 3*D* *top-left*).

**Figure 4:**
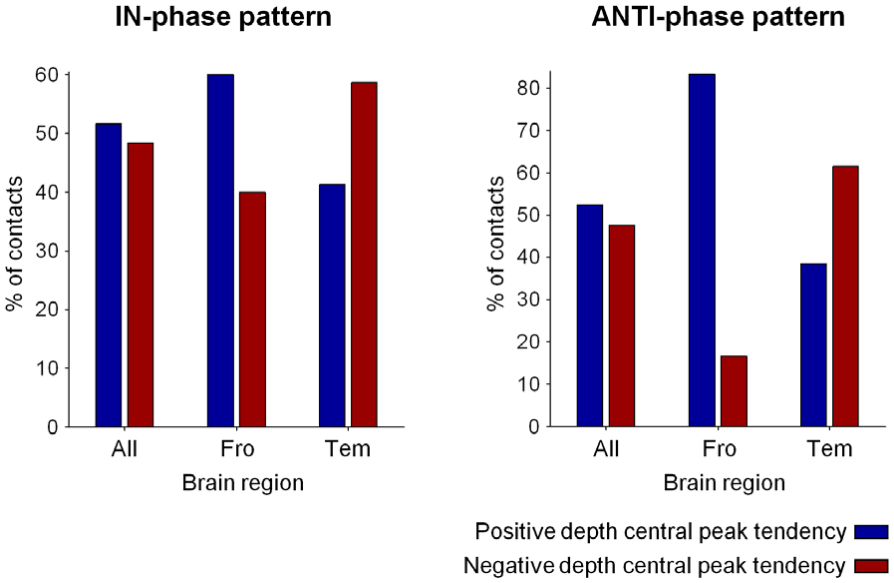
Percentage of intracranial contacts presenting depth-positive (*blue bars*) or depth-negative components (*red bars*) during the IN-phase (*left*) and ANTI-phase (*right*) patterns for 15/19 and 7/19 subjects respectively, see the text. This analysis was performed by taken into account all contacts ( “ALL”) or only contacts located in the frontal (Fro) and temporal (Tem) cortex.

Recorded intracranially, slow waves showed depth-positive or depth-negative components (see Figure 3*A and 3B*), reflecting respectively inward currents in the superficial layers and outward currents in the deep cortical layers [17]. In order to assess whether IN-phase and ANTI-phase patterns were related to particular depth-components of the slow waves, we examined the value of the central peak of the intracranial slow wave associated to each pattern (Figure 1). In particular, for all slow waves associated with IN-phase and ANTI-phase patterns (from 15/19 and 7/19 subjects respectively, see above) we investigated if there was a systematic reverse of polarity between scalp and intracranial waves. No significant relationship was found between the polarity reversal of the intracranial slow wave and the corresponding phase of appearance of gamma events (Figure 4).

**Figure 5:**
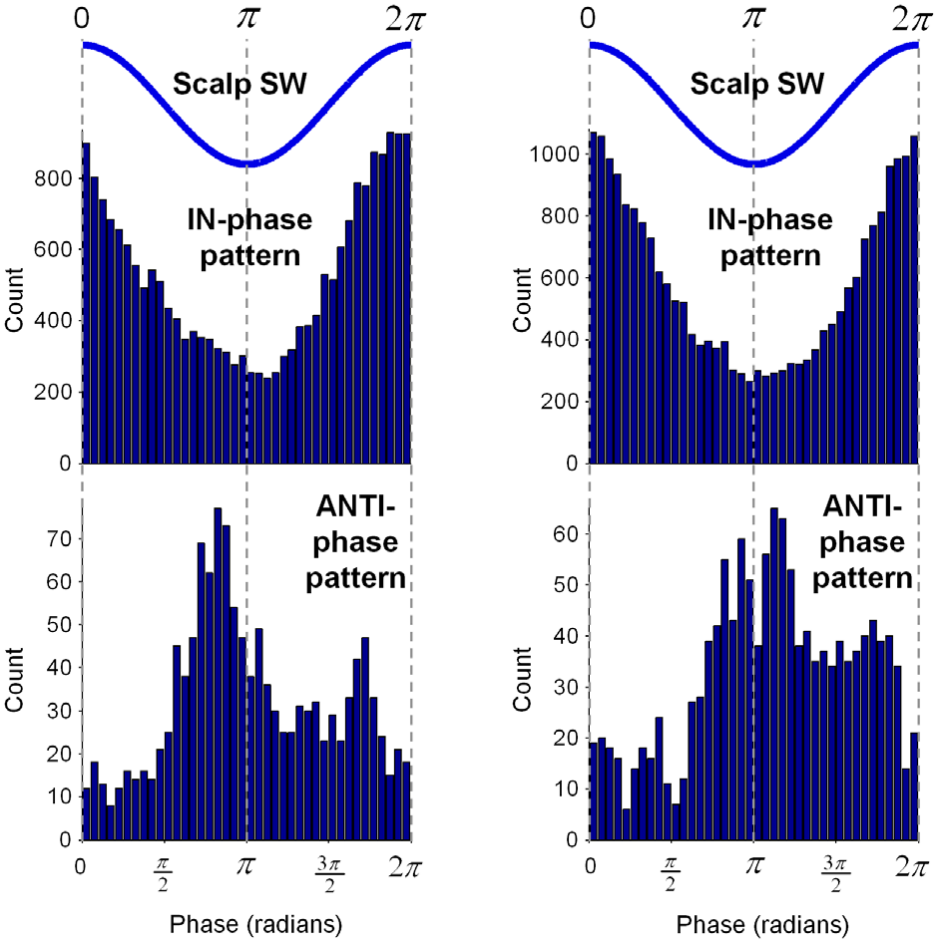
Histograms presenting the distribution of the preferred phases for all contacts associated with IN-phase (122 from 15 subjects, *top* histogram) and ANTI-phase (21 from 7 subjects, *bottom* histogram) patterns, for the first detection time (*left*) and the maximal amplitude time (*right*). Phases of the corresponding SW are given for reference (blue lines on *top*).

We next analyzed the temporal occurrence of IN-phase and ANTI-phase patterns relative to the central peak of the scalp slow wave (time 0 in Figures 3*C* *bottom* and 3*D bottom*). We observed that events associated with the IN-phase pattern preferentially appeared at around −0.5 seconds and 0.28 seconds of the slow wave cycle, and tended to be absent around −0.165 seconds (Figure 3*C* *bottom*). In contrast, events associated with the ANTI-phase pattern preferentially occurred at around −0.05 seconds of the slow wave cycle (Figure 3*D* *bottom*). To further investigate the phase relationship between slow waves and gamma oscillations, we distinguished two different moments of the gamma events: first, the onset time at which the event was first detected (*τ_o_* in Figure 1) and, second, the time at which the event reached its maximal amplitude (*τ_m_* in Figure 1). In order to quantify whether these events tended to systematically occur at a particular phase of the slow wave, we used a circular statistical test to assess the nonuniformity of the corresponding phase distribution across all the detected slow waves (see *Materials and Methods*). Quantitative assessment revealed that, from all contacts associated respectively with IN-phase (122 from 15 subjects) and ANTI-phase (21 from 7 subjects) patterns, 44% (54/122) and 52% (11/21) presented statistically significant phase-locking (*p*<0.01, Rayleigh test for circular uniformity) when considering the event's onset time, and 52% (64/122) and 52% (11/21) when considering the event's maximal amplitude time (Figure 5). In accordance with the distributions in time, relative to the central peak of the slow wave, the preferred phases calculated for the onset times and maximal amplitude times respectively, were obtained for the IN-phase and the ANTI-phase patterns at 6.13 radians (∼0.5625 sec) and 3.05 radians (∼−0.015 sec), and at 0.1 radians (∼−0.5675 sec) and 3.8 radians (∼0.125 sec) for the same, respective patterns. Interestingly, for the onset time of ANTI-phase patterns, the phases of the slow wave tended to be highly concentrated in a sharp peak, whereas the phases of maximal amplitude time tended to lie in a wider interval.

**Figure 6:**
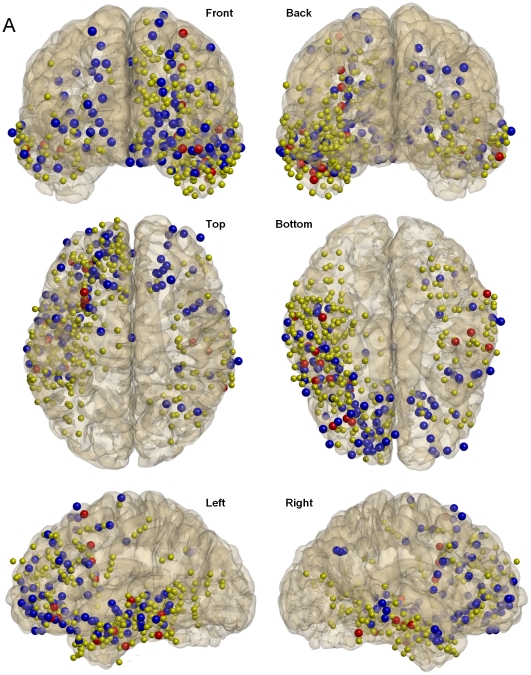
Spatial distribution of phase-modulated gamma patterns. Locations of all intracranial contacts associated with IN-phase (*blue balls*) and ANTI-phase (*red balls*) patterns for 11 subjects for which Talairach coordinates could be successfully estimated are superimposed on a cortical reconstruction of one subject. Yellow balls correspond to the locations of all analyzed contacts for the corresponding 11 subjects (418). From left to right and from top to bottom, views are presented from the following planes: frontal-coronal, back-coronal, top-axial, bottom-axial, left-sagital and right-sagital.

The spatial distribution of the electrode contacts related to each of these two patterns revealed that phase-modulated gamma patterns were strongly expressed in the frontal and temporal lobes (Figure 6). In particular, 39% and 37% of electrodes associated with the IN-phase pattern were localized in the frontal and temporal lobes respectively while, for the ANTI-phase patterns, 29% and 62% of electrodes were associated respectively with the frontal and temporal lobes. This suggests a tendency for the ANTI-phase pattern to be more expressed in temporal regions while the IN-phase pattern was more homogeneously distributed in any of these two lobes (percentages of electrodes in other regions are not presented because the involved contacts were less than 10%, partially due to the fact that most of the contacts were located in frontal and temporal regions, see Table S1).

We further examined if the sites expressing IN-phase or ANTI-phase patterns were related to pathological areas. We observed that most of the contacts associated with both modulation patterns were neither located in ictal nor inter-ictal regions. More precisely, from all contacts associated with IN-phase patterns, 84% and 80% were not located respectively in ictal or inter-ictal zones; For ANTI-phase patterns, 90% and 71% of contacts were not correspondingly associated with ictal or inter-ictal zones (Tables S2 and S3).

### Short-range intracortical coherence of gamma oscillations during SWS

**Figure 7:**
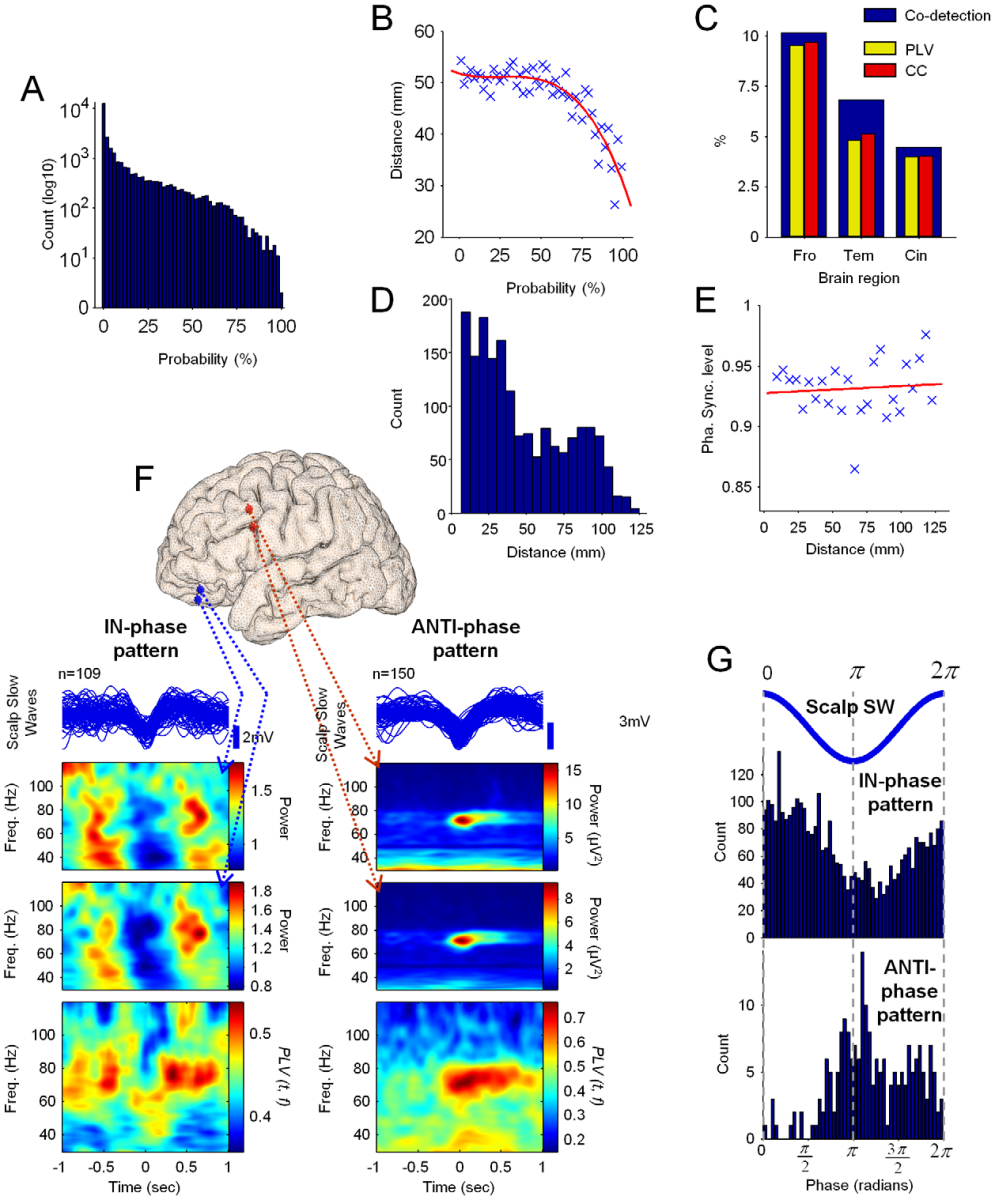
Synchronization of gamma events occurring at different intracranial contacts during slow waves. (**A**) Distribution of *co-detection* probability for all analyzed contacts (n = 20 subjects). (**B**) Distribution of the *co-detection* probability vs. the distance between pairs of contacts and the corresponding regression curve (red line, n = 11 subjects). (**C**) Percentages of contact pairs presenting a *co-detection* probability ≥50% and located in frontal, temporal and cingulate cortex (wide blue bars in background), and corresponding percentages having significant PLV and CC values (yellow and red bars in foreground respectively, n = 20 subjects). (**D**) Histogram of the distance between pairs of contacts for all cases presenting *co-detection* probability ≥50% and statistically significant PLV (n = 11 subjects). (**E**) Plot of the distance between contact pairs vs. the PLV level with the corresponding regression curve (*red line*) (n = 11 subjects). (**F**) Examples of contact pairs presenting statistically significant phase synchronization modulated by IN-phase and ANTI-phase patterns. From top to bottom: superimposed scalp slow-waves associated with the first contact in the pair (see below), aligned around the central peak; Average time-frequency representations of the power for the first and second contacts; Phase-locking value for the phase difference between both contacts along all slow waves. Pairs of contacts (*first* and *second*) were implanted for subjects *P7* and *P13* respectively in the following locations: orbital sulcus and olfactory sulcus (*left*), frontal superior sulcus and frontal inferior sulcus (*right*). The distance between pairs of contacts shown in both examples was 10 mm. (**G**) Histograms presenting the distribution of the preferred phases for all cases presenting statistically significant phase-sycnhronization across all slow waves and associated with IN-phase (*top* histogram) and ANTI-phase (*bottom* histogram) patterns. Phases of the corresponding SW are given for reference (blue line on top) (n = 8 subjects).

The synchronization properties of gamma events occurring at different intracranial contacts during slow waves were evaluated. We first estimated the *co-detection probability*, i.e. the probability of detecting a gamma event in one contact at a specific time, given that an event has been also detected in another contact, for the same time and for the same frequency band (see *Materials and Methods*). The distribution of this probability revealed an exponential-like decrement (Figure 7*A*) which was best fitted to a gamma distribution (4.79×10^−5^ mean squared error; shape and scale parameters: k = 0.88 and θ = 23.26). This observation implied that the probability to find a co-activation between two arbitrary channels was very low despite the fact that gamma oscillations emerged in a large number of investigated individual channels. This obtained tendency was further confirmed by the relation between the *co-detection probability* and the distance between contacts, calculated via their Talairach coordinates in the 3D space (n = 11 subjects, Figure 7*B*). We found that the *co-detection probability* of gamma oscillations fell off quickly with distance, suggesting that gamma co-activations can only be frequently observed over short-distances, therefore within a single cortical area or between adjacent cortical areas. The slope of the decrement was estimated to be around −0.41 mm per probability percentage, meaning, for example, that the probability for a given event to be detected at the same time in two contacts would be 25% lower if the separation between them were increased by 10 mm. The cortical distribution of channels presenting a *co-detection* probability over 50% revealed that the frontal lobe had a greater tendency to express gamma co-activations, followed by the temporal lobe and the cingulate cortex (Figure 7*C*, *blue bars*). In contrast, the parietal, occipital and insular cortices only displayed a very low tendency of gamma co-activations.

We next examined whether gamma activities that co-occurred in space at two different places were also oscillating in phase (i.e. were phase-locked). For this, we selected all channels with a *co-detection probability* over 50% (2121 pairs of contacts from 14 subjects and from a total of 28417 pairs over all analyzed frequency bands in the gamma range) and we calculated the windowed phase-locking value (*PLV*) and the cross-correlation coefficient (CC), both quantities giving values varying from 0 to 1, where 1 means perfect synchrony (see *Materials and Methods*). In a large number of cases, significant synchronization could be identified during gamma co-activations by both measurements (*p*<+0.001, two-tailed permutation-test; for *PLV* and *CC*, in 83.5% and 86% of the cases respectively). Similarly to gamma co-activations, we found that the number of synchronous pairs fell off quickly with distance (Figure 7*D* and Figure S2 *A*). Nevertheless, in contrast to gamma co-activations, the strength of the synchronization did not decrease with the distance, suggesting that strong synchrony could happen, with a low probability, between two widely separated contacts (Figure 7*E* and Figure S2 *B*). The cortical distribution of channels presenting significant synchronous activities (for both *PLV* and *CC*) revealed that the frontal lobe had a greater tendency to express more gamma synchronization followed by the cingulate and the temporal ones (Figure 7*C* *, yellow and red bars*).

We finally examined the relationship between the phases of the slow wave and the co-activations of gamma oscillations. In particular, from all channels presenting significant synchrony, we evaluated whether co-activations tended to systematically appear at a particular phase of the slow wave (see *Materials and Methods*). From all analyzed co-activations, we found that only 2.8% presented a systematic temporal relationship with the slow wave (*p*<0.01, Rayleigh test for circular uniformity). Examples of contact pairs presenting this infrequent phenomenon are shown in Figure 7*F*. For these pairs of contacts, we estimated the phase-locking of the gamma oscillations calculated across all corresponding slow waves. As illustrated in these examples and observed most of the time (65.4%, representing pairs of contacts from 8 subjects), the phasic modulation of gamma synchronization corresponded to IN-phase and ANTI-phase patterns (Figure 7*G*). The preferred phases together with the preferred times were estimated respectively to 0.74 radians (∼−0.4475 sec) and 3.87 radians (∼0.14 sec) for IN-phase and ANTI-phase patterns.

## Discussion

The main observations of our study can be summarized as follows: 1) Gamma oscillations in the range 30–120 Hz were observed during SWS in the majority of investigated cortical LFPs. They were characterized by oscillations in narrow frequency bands in the low (∼30–50 Hz) and high gamma (∼60–120 Hz) ranges. 2) Different patterns of phasic activation of gamma oscillations could be identified: IN-phase gamma patterns tended to appear around the positive phase of the scalp SW. They were strongly expressed in the frontal and temporal cortical regions and were, in most of the cases, constituted by narrow band frequencies distributed over the whole gamma range from 30 to 120 Hz. In contrast, ANTI-phase patterns tended to appear during the negative phase of the scalp slow wave. They were more expressed in temporal regions and had only a few dominant peaks in the high gamma range (around 70 and 95 Hz). 3) The spatial coherence of gamma events occurring at different intracranial contacts during slow waves was only local and fell off quickly with distance.

A first novel finding of our study was that spontaneous gamma oscillations from 30 to 120 Hz, recorded at the macroscopic EEG level, were strongly expressed during deep sleep in the human cortex. This may be surprising because faster rhythmic EEG activity were generally reported in relation to waking functions such as sensory perception, attention or memory [18]–[20]. Furthermore, several previous EEG studies have reported that gamma activity is highest during wakefulness and REM, and lowest during SWS, in both humans [21], [22] and animals [23]. As mentioned before [11], this discordance could be explained by the fact that, in those previous studies, fast oscillatory activities were estimated through an average over several minutes of recording which is not an appropriate methodology for the detection of transient events lasting only few hundred milliseconds and separated by long periods of silence. Our results are nevertheless in agreement with LFPs observations in sleeping or anesthetized animals reporting activations in the beta/low gamma and high gamma ranges during the depolarization phase of the slow wave [3]–[7], [24]. Furthermore, our observations are consistent with a recent microelectrode study reporting that distinct narrow band oscillations in the low (40–80 Hz) and high (80–120 Hz) gamma ranges were locally expressed in the LFPs of the human cortex during SWS [11]. In accordance, we confirmed with clinical macroelectrodes in the human cortex that different discrete spectral peaks can be consistently identified in the low and high gamma range. On the basis of these observations, we can postulate that our recorded gamma oscillations reflect the same local, highly synchronous field activities as those recorded with microelectrodes. The possibility to record these oscillations with intracranial macroelectrodes of large surface area greater than 1 mm^2^ suggests that an activity of ∼100 mm^3^ of brain tissue should be involved here [25].

As previously shown [26], [27], minimal eye movements (so-called microsaccades) can induce gamma activities in scalp EEG but also in intracranial recordings. We think however that the gamma activities reported in our analyses are unlikely the product of such a kind of artifacts but instead the result of a physiological cortical activity. Arguments in favor of this are: (1) we analyzed only periods during NREM, and in particular during slow wave sleep stages where the eye movements (rolling eye balls) are minimum compared to wake or REM sleep stages; (2) while, when they appeared, gamma-activities were clearly visible on intracranial contacts, they were absent at the scalp level; (3) gamma activities reported here were found to be modulated by the slow oscillations, known to generate no phasic motor events.

A second novel observation of the present study was the existence of two gamma patterns in the human cortex defined by their relations with the phases of the cortical slow oscillations. In most of the cases, gamma oscillations were correlated with positive peaks of scalp slow waves, confirming previous animal data suggesting that gamma oscillations occur during the active component of the slow wave (‘UP’ state). This observation of enhanced gamma activities during surface-positive components of slow oscillations is also consistent with the grouping of spindle and beta activities reported for human scalp EEG [13], [14]. This depolarization phase is associated with increased cortical firing, which drives the generation of spindle, beta and gamma oscillations in thalamo-cortical feedback loops [3]. In addition, we also reported ANTI-phase gamma events, having distinctive frequency/spatial properties and appearing preferentially during surface-negative components of slow waves corresponding to cortical cellular hyperpolarization. These ANTI-phase patterns had onset times highly concentrated around the negative peak of the scalp slow waves, in contrast to the phases of their maximal amplitude times, more scattered over a wider interval. This further suggests that ANTI-phase gamma events were initiated at the beginning of the DOWN state, known to be precisely synchronized [28]. Interestingly, cellular activities and gamma oscillations were already reported in the rat hippocampus to emerge during the neocortical-paleocortical DOWN states [5]. One explanation for our reported ANTI-phase patterns, particularly expressed over the temporal cortex, is that hippocampal projections to the cortex drive fast rhythmic post-synaptic EPSPs during the hyperpolarizing (surface negative) phase of the slow wave, associated with cortical firing silence. The global lack of correlation between higher density of gamma oscillations and epileptogenic tissue provides some reassurance, albeit indirect, that gamma activity was not mainly caused by epilepsy. Nevertheless, it cannot be fully excluded that ANTI-phase patterns were not generated by aberrant synchronization remote from chronic epileptic circuits. Additional research is here needed to precise the neuronal origins of this new type of pattern.

Thanks to the relatively large sample size as well as to a broad spatial sampling, we were able to evaluate in our study the intracranial coherence of gamma oscillations over a comprehensive extent of the human cerebral cortex. According to our observations, gamma oscillations were frequently phase-locked over short-distances, but this synchronization decreased rapidly in space. This local spatial coherence between close-by electrodes could be explained by the large surface area of the used macroelectrode that may record partly overlapping source distributions. In the same way, it has been reported in felines that the coherence at approximately zero phase lag between activities in high-frequency ranges was only observed for cortical sites located within few millimeters of distance (<5 mm) [3]. Our findings are also consistent with other studies in humans that were not able to demonstrate gamma band coherence at large distance (greater than 14 mm) between intracranial EEG sites [29]. This short-range spatial confinement of coherent fast rhythms is consistent with previous observations during wakefulness reporting that fast oscillations are synchronized locally in both space and time, as shown by the very restricted cortical areas and time windows in which coherent fast oscillations appear [30].

What could be the function of these gamma patterns? As proposed before, the network dynamics during active states of deep sleep have been proposed to be equivalent to those observed during the waking state [31], [32]. In agreement with this proposal, our observations of gamma during SWS are very similar to gamma responses induced by a variety of waking tasks reflecting an increased alertness and also recorded with intracranial EEG [19], [33], [34]. Furthermore, activation patterns during SWS are also known to be close to the waking default mode network (DMN) [35], [36]. Following this similarity, it has been proposed that gamma oscillations during SWS may reflect recalled events experienced previously [11], [37]. Interestingly, recent intracranial EEG evidences suggested that all DMN areas displayed transient increases and decreases of broadband gamma (60–140 Hz) power during goal-directed behavior [38]. Therefore, gamma oscillations during SWS could recurrently restore an intrinsic form of large-scale brain dynamics, typical of wakefulness, and trigger over time an activity-dependent reinforcement pathway like LTP, leading to a progressive consolidation of local cortical circuits [39], thus complementing the roles of gamma oscillations in encoding and retrieval of memory traces during wakefulness [40], [41]. Indeed, as previously reported [11], these cortical gamma oscillations may be also triggered by hippocampal ripples-sharp waves and may coordinate, at the cortical level, the reactivation of hippocampus-dependent memories [42], [43]. In this context, as it has been proposed by others [5], while gamma oscillations during “UP” states (IN-phase pattern) may facilitate the hippocampal-neocortical transfer of previously coded information via parahippocampal pathways [11], neocortical gamma oscillations during “DOWN” states (ANTI-phase pattern) may reflect an intrinsic hippocampal replay and a modification of intrahippocampal and entorhinal cortical connectivity. Additional research is here justified to clarify the role of IN-phase or ANTI-phase gamma events in the supposed iterative mechanisms of memory reprocessing during SWS. It is anticipated that further insights into the role of gamma activities during sleep will accrue from studying phenomena such as lucid dreaming [44] and sleep-state misperception [45], in which patients report the subjective experience of wakefulness during polysomnographically defined sleep.

We have reported here the presence of intracranial gamma oscillations in epileptic patients recorded during seizure-free periods and we have shown that these oscillations coincide with non epileptic activities such as slow sleep oscillations. Nevertheless, epileptic oscillations in the same frequency range are well known in intracranial EEG recordings of the epileptic regions of patients with temporal and extra-temporal epilepsy, at seizure onset [46], [47] or throughout the interictal period [48]. Furthermore, as already reported in patients with frontal lobe seizures [49], epileptic gamma oscillations appear to be closely related to sleep, especially in the prefrontal and orbitofrontal cortex. In addition to these observations, experimental models have led to the proposal that these fast oscillations could be involved in the initiation of seizures [50]. This raises the question about the relations between physiological and epileptic gamma oscillations. From our point of view [11] and as also suggested by other authors [48], the increased high frequency activity seen at the onset of some seizures could be the result of an aberration of the same physiological mechanisms underlying gamma activations in SWS. Indeed, strong gamma oscillations may be driven in the epileptic cortex by transient paroxysmal neuronal depolarization, in a similar way than the strong potential fluctuations which occur during the normal sleep. Furthermore, epileptic processes are well-known to be characterized by a general tendency of hypersynchronization of normal oscillations, as seen for example in the epileptic facilitation of sleep spindles [51] or ripples [52]. This aberrant hyper-expression may possibly imply the same physiological mechanisms underlying memory consolidation, leading to a progressive reinforcement of local epileptogenic circuits [48].

## Materials and Methods

### Subjects and recordings

We analyzed twenty subjects (10 females; age range, 18–49 years; mean age, 32 years) with refractory partial epilepsy undergoing presurgical evaluation, hospitalized between February 2002 and July 2007 in the epilepsy unit at the Pitié-Salpêtrière hospital in Paris. Each patient was continuously recorded during several days (duration range, 9–20 days; mean duration, 15 days) with intracranial and scalp electrodes (Nicolet acquisition system, CA, US). Both scalp and depth EEG data were continuously recorded at a sampling rate of 400 Hz. The placement of scalp electrodes was adapted to each patient in order to facilitate the implantation of intracranial electrodes but the montage always involved electrodes at T9, TP9, T10, TP10, FPZ, FP1 and FP2 locations. Depth electrodes were composed of 4 to 10 cylindrical contacts 2.3-mm long, 1-mm in diameter, 10-mm apart center-to-center, mounted on a 1 mm wide flexible plastic probe. Subdural electrodes were strips with 4 to 8 one-sided circular contacts, 2.3 mm in diameter and with a center to center separation of 10 mm. Pre and post implantation MRI scans were evaluated to anatomically and precisely locate each contact along the electrode trajectory. Talairach coordinates of intracranial contacts could be successfully estimated for 11 subjects through BrainVisa/Anatomist (http://brainvisa.info/index_f.html). The placement of electrodes within each patient was determined solely by clinical criteria; however, the routine clinical use of broad anatomical coverage for intracranial recordings provided a large sample of electrophysiological data from tissue outside of the epileptogenic zone (Table S1). Common average reference montage was chosen as our previous work on intracranial high-frequency activities [25]. This montage was chosen in order to avoid the introduction of unrelated information from the two referential time-series into the bipolar time-series, or from the removal or alteration of information common to the two referential time-series in the bipolar time-series. Noisy electrodes and those presenting high epileptic activity were excluded from the average by visual inspection. Digital bandpass filtering between frequencies *f_1_*−*f_2_* was, in all cases, implemented through a forward-backward digital infinite impulse response (IIR) type II Chebyshev filter (*passband*: *f_1_*≤*f*≤*f_2_* Hz, attenuation ≤1 dB, monotonic; *stopband*: *f*≤(*f_1_*−*k*) or *f*≥(*f_2_*+*k*) Hz, where *k* = 0.5 for *f_1,2_*≥1 Hz or *k* = 0.05 otherwise, attenuation ≥100 dB, equiripple). Electrical line noise at 50 Hz was suppressed by a bandstop filter of the same type. All analyses were implemented in MATLAB® 7.5 (The MathWorks™, MA, USA). All patients gave their written informed consent and procedures were approved by the local ethical committee (CCP).

### Sleep studies

Two seizure-free nights with at least two complete sleep cycles were chosen from all subjects. Each night was scored for sleep stages using the software Somnologica™ Studio (Embla Systems, Inc, CO, USA) and visually confirmed by a time-frequency analysis.

### Slow waves detection

Slow waves were first detected from a single scalp electrode and only from segments during NREM sleep. Since not all subjects shared exactly the same number of scalp electrodes, FP1 was chosen for the analysis because it was systematically recorded. For the automatic detection of the slow waves, the signal was first down-sampled to 40 Hz and then bandpass-filtered between 0.1–4 Hz. The criteria for the detection of the slow waves were similar to those used in [53] and [16]: (1) a negative wave between two succeeding zero-crossings separated by 0.125–1 sec and presenting only one main peak ≤−80 µV and, optionally, other negative peaks not exceeding 50% of the main one (in absolute value); (2) a subsequent (or antecedent) positive wave between two succeeding zero-crossings separated by 0.125–1 sec; (3) a negative-to-positive (or positive-to-negative) peak-to-peak amplitude ≥140 µV (see Figure 1). In order to select only large-extended slow waves, an additional criterion was used to confirm the simultaneous presence of the slow components on intracranial contacts. According to that, we only kept waves for which the average of all intracranial negative peaks (when presented in at least 30% of the total number of contacts) during the scalp positive peak was ≤−60 µV. Finally, we rejected slow waves presenting interictal epileptic discharges inside a window of 2 seconds around the minimal scalp negative peak.

### Automatic detection of gamma oscillations

An automatic detection of high-frequency events was performed separately for each intracranial contact and for the whole sleep-wake cycle and independently of the presence of slow waves. In order to detect oscillations, the whole gamma range was subdivided in consecutive sub-bands of 20-Hz each (*f_2_*−*f_1_ = 20 Hz*, *f_2_>f_1_*), from 30 to 120 Hz. For each one of these sub-bands, oscillations were detected according to a similar procedure than those presented in [15]: First, the envelope of the bandpass-filtered signal was obtained via the Hilbert transform. Then, a threshold for detection was defined consisting on all successive envelope values with amplitudes >3σ above the mean amplitude of the envelope signal (the mean and the standard deviation (σ) computed from free-of-artifacts periods, selected along the whole night by visual inspection). From all detected events, we selected only events having more than 6 oscillatory cycles (calculated from the mean frequency (*f_1_*+*f_2_*)/2 of the current frequency band), and presenting more than 5 local maxima in both original and filtered signals (see Figure 1).

### Oscillation analysis

The central frequency of each detected oscillation was determined by the frequency at which a peak appeared in the power spectral density (PSD) obtained via the Burg method. The corresponding signals were zero-padded to the next higher power of two, but in all cases the resulting length was ≥512 points. The order of the autoregressive model was set to 10.

### Time-frequency representations

Time-frequency transformations for power and phase estimations were obtained through a Gabor wavelet, implemented with a modulated Gaussian window [54]. The number of cycles of the wavelet was set to 5.

### Phasic modulation of gamma oscillations by the slow waves

In order to determine possible patterns of phasic modulation by the slow waves, we estimated separately for each intracranial contact the distribution in time of gamma events detected inside a window of 2 seconds around the minimal scalp negative peak of the slow waves. Only intracranial contacts presenting slow waves were analyzed by requesting that the absolute value of the cross-correlation coefficient between the scalp slow wave and the related intracranial one was ≥0.6 since these values allowed the simultaneous analysis of scalp and intracranial slow wave activity. For all these investigated intracranial contacts, these distributions were then grouped in different patterns following a criterion of similarity, consisting in grouping the most similar among them. For this, we calculated the cross-correlation between all possible distributions and we considered a group when all the cross-correlation coefficients between their distributions had a minimal value of 0.76, corresponding to the value at which the number of elements inside one of the groups became stable.

### Co-detection probability and distribution fitting

For every pair of intracranial contacts, we estimated the probability of detecting a gamma event in one contact given that an event, in the same frequency band and for the same time, has been also detected in another contact. Co-detection was defined when both events had an overlapping of at least 50% respect to the total event duration (Δ*τ_w_* in Figure 1). Probability estimation was performed only for frequency bands presenting at least 25 events. In order to fit the statistical distribution given by the *co-detection* probability, we tested the following known distributions: Exponential, Poisson, Gamma, Chi-squared, Rayleigh and Weibull.

### Synchronization between two gamma oscillations

Synchronization between pairs of *co-detected* gamma events was estimated by the cross-correlation coefficient (CC) and the windowed phase-locked value (PLV). Their analysis followed two steps: First, pairs of signals were narrow-band filtered in the corresponding co-detection frequency band. Second, the following two measures were calculated: 1) CC: For the pair of filtered signals, the cross-correlation coefficient was estimated by *CC* = *C*(*s_1_*, *s_2_*)/(*C*(*s_1_*, *s_1_*) *C*(*s_2_*, *s_2_*))^1/2^, where *C* corresponded to the covariance function (at zero-lag) and *s_k_* for *k* = 1,2 to signal *k*. Thus, *C*(*s_1_*, *s_2_*) corresponded to the cross-covariance at zero-lag between the pair of signals in the *co-detected* event and *C*(*s_1_*, *s_1_*) and *C*(*s_2_*, *s_2_*) to the variance for each signal respectively. 2) PLV: This analysis was introduced to overcome some limitations of correlation methods which do not disentangle amplitudes and phases [55]. The phases of both filtered signals were calculated via the Hilbert transform and the difference between them was then calculated. The phase locking was next estimated by 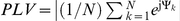, where Ψ*_K_* is the phase difference (mod 2π) for the corresponding *k* time and *N* the total number of phase difference values inside the time window corresponding to the co-detection event (Δ*τ_w_* in Figure 1). In order to further test for statistical significance, surrogate data was generated from each one of both measurements and a permutation test was then performed [56].

### Phase-locking of gamma activations and slow waves

In order to examine the relationship between the phases of the slow wave and the activation or the co-activation of gamma oscillations, we used following procedure: First, the phases of the slow waves were estimated through the Hilbert transform of the filtered signals in the low frequency band (0.1–4 Hz). Second, the phase value ϕ*_K_* (mod 2π) of the slow waves corresponding to the detection or the co-detection times were computed (depending on the analysis, it could be *τ_o_* or *τ_m_* in Figure 1). Finally, from the first trigonometric moment defined by 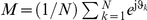, a phase modulation index was estimated across all corresponding slow waves by the modulus of *M*, where N was the total number of phase values. Given the Rayleigh statistic Z = N |*M*|^2^, the probability that the null hypothesis of sample uniformity holds was given by P = *e* ^−Z^ [1+(2Z−Z^2^)/(4N)−(24Z−132Z^2^+76Z^3^−9Z^4^)/(288N^2^)] [57]. The preferred phase was estimated by the phase of *M* and the corresponding preferred time was approximated by a slow wave of frequency 0.85 Hz.

### Phasic modulation of gamma phase synchronization by the slow waves

In order to examine the relationship between the phases of the slow wave and the phase synchronization between gamma oscillations, we used a similar procedure than those previously used [55]: First, the phases of both signals were calculated through a Gabor wavelet between 30–120 Hz and inside the 2-seconds window around the scalp negative peak of the corresponding slow waves. The difference between corresponding computed phases was then obtained. Then, the phase locking across all slow waves was estimated for the time *t* and the frequency *f* by 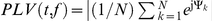, where Ψ*_K_* was the phase difference (mod 2π) for the corresponding slow waves of index *k* and *N* the total number of slow waves.

## Supporting Information

Figure S1 Histograms presenting the frequency distribution of detected gamma events associated with IN-phase (*left*) and ANTI-phase (*right*) patterns, for the examples presented in Figures 3*A and 3B* respectively.
(TIF)

Figure S2 Synchronization of gamma events as estimated through the cross-correlation coefficient (CC). (**A**) Histogram of the distance between pairs of contacts for all cases presenting *co-detection* probability ≥50% and statistically significant CC (n = 11 subjects). (**B**) Plot of the distance between contact pairs vs. the CC level with the corresponding regression curve (*red line*) (n = 11 subjects).
(TIF)

Table S1 Depth electrodes implantation data. Contacts in the epileptic ictal zone corresponds here to contacts associated with seizure onsets. Contacts in the epileptic interictal zone corresponds here to contacts associated with epileptic spikes during interictal periods. Contacts between two adjacent regions were considered as half in each one.
(DOC)

Table S2 Regional distribution of intracranial contacts related to IN-phase pattern.
(DOC)

Table S3 Regional distribution of intracranial contacts related to ANTI-phase pattern.
(DOC)
