# Supplementary figures and images for: Human Gamma Oscillations during Slow Wave Sleep
Source: PLoS One. 2012 Apr 4;7(4):e33477. doi: 10.1371/journal.pone.0033477 (PMC3319559; doi:10.1371/journal.pone.0033477)

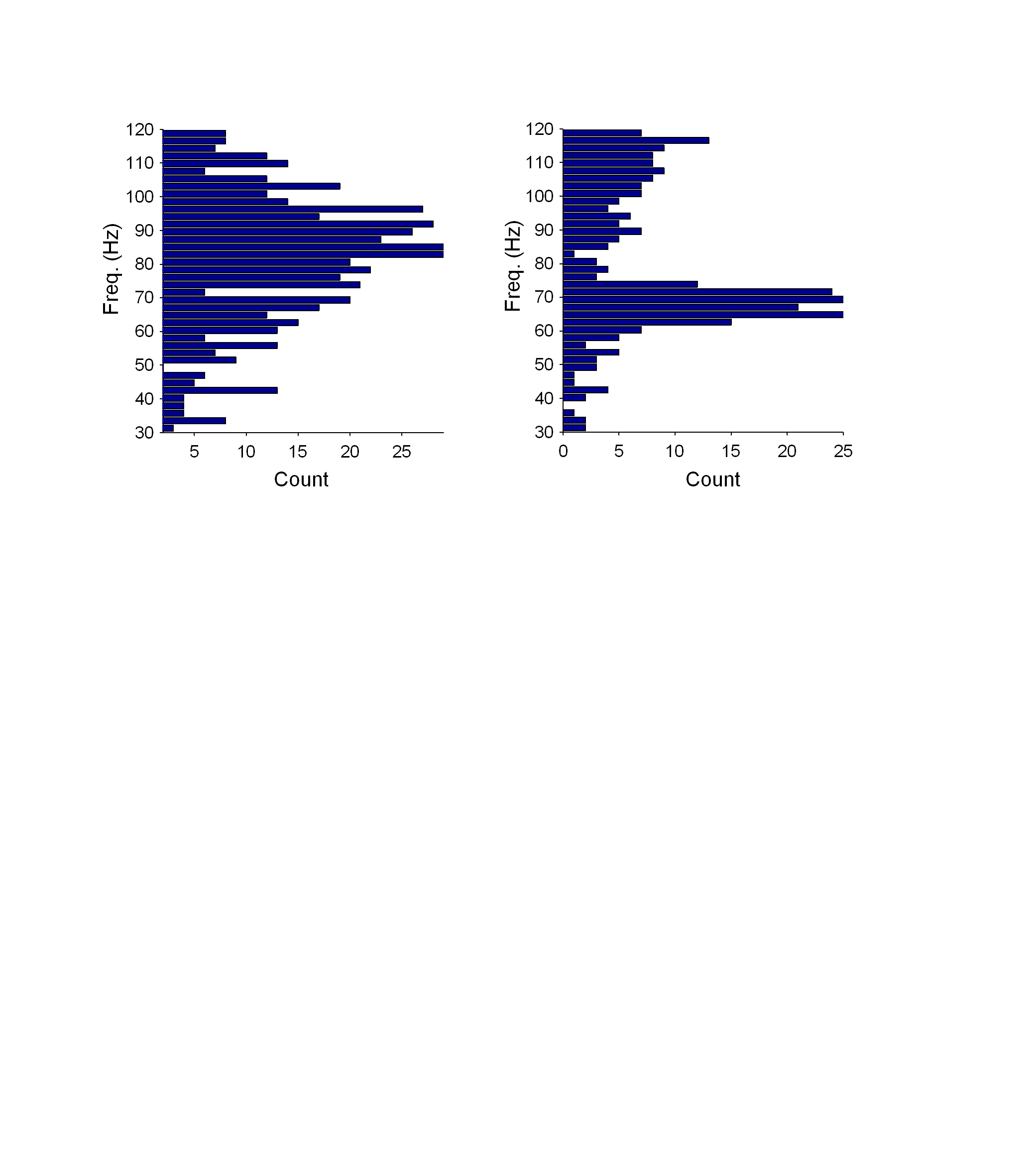

Supplement: Figure S1 — Histograms presenting the frequency distribution of detected gamma events associated with IN-phase (left) and ANTI-phase (right) patterns, for the examples presented in Figures 3A and 3B respectively. (TIF) [file pone.0033477.s001.tif]

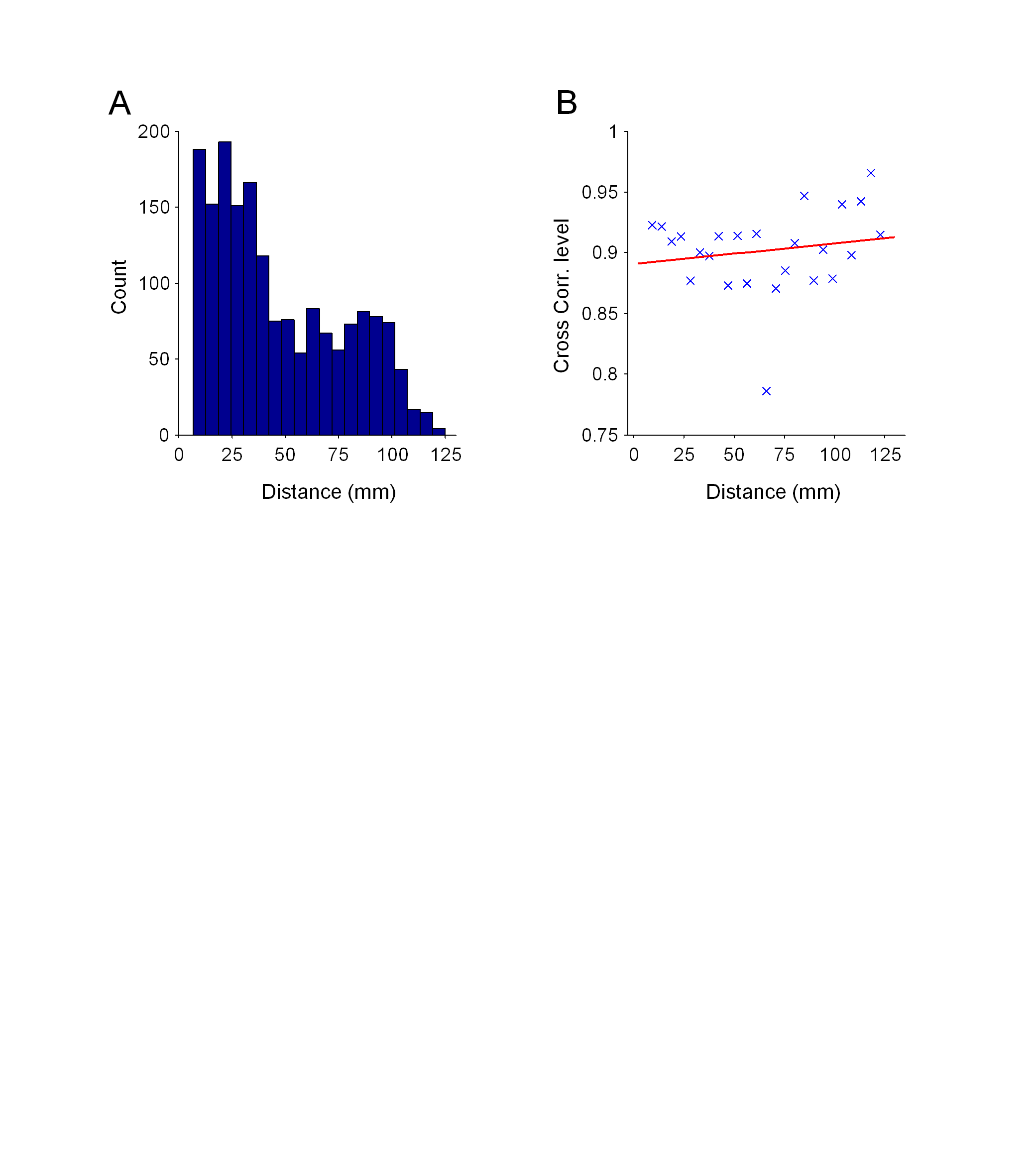

Supplement: Figure S2 — Synchronization of gamma events as estimated through the cross-correlation coefficient (CC). (A) Histogram of the distance between pairs of contacts for all cases presenting co-detection probability ≥50% and statistically significant CC (n = 11 subjects). (B) Plot of the distance between contact pairs vs. the CC level with the corresponding regression curve (red line) (n = 11 subjects). (TIF) [file pone.0033477.s002.tif]
