# Supplementary material for: Human Gamma Oscillations during Slow Wave Sleep
Source: PLoS One. 2012 Apr 4;7(4):e33477. doi: 10.1371/journal.pone.0033477 (PMC3319559; doi:10.1371/journal.pone.0033477)
Supplement: Table S1 — Depth electrodes implantation data. Contacts in the epileptic ictal zone corresponds here to contacts associated with seizure onsets. Contacts in the epileptic interictal zone corresponds here to contacts associated with epileptic spikes during interictal periods. Contacts between two adjacent regions were considered as half in each one. (DOC) [file pone.0033477.s003.doc]

Table S1.

^a^Frontal; ^b^Parietal; ^c^Occipital; ^d^Temporal; ^e^Cingulate; ^f^Insular; ^g^Striatum.
