# Supplementary material for: Human Gamma Oscillations during Slow Wave Sleep
Source: PLoS One. 2012 Apr 4;7(4):e33477. doi: 10.1371/journal.pone.0033477 (PMC3319559; doi:10.1371/journal.pone.0033477)
Supplement: Table S2 — Regional distribution of intracranial contacts related to IN-phase pattern. (DOC) [file pone.0033477.s004.doc]

Table S2.

^a^Frontal; ^b^Parietal; ^c^Occipital; ^d^Temporal; ^e^Cingulate; ^f^Insular; ^g^Striatum.
